# Dynamic context-dependent regulation of auxin feedback signaling in synthetic gene circuits

**DOI:** 10.1073/pnas.2309007120

**Published:** 2023-10-09

**Authors:** Merisa Avdovic, Mario Garcia-Navarrete, Diego Ruiz-Sanchis, Krzysztof Wabnik

**Affiliations:** ^a^Centro de Biotecnología y Genómica de Plantas (CBGP), Universidad Politécnica de Madrid (UPM)-Instituto Nacional de Investigación Agraria y Alimentaria (INIA/CSIC), 28223 Madrid, Spain

**Keywords:** auxin signaling, feedback regulation, cellular information processing, synthetic biology, dynamic environment

## Abstract

Phytohormone auxin plays a key role in regulating plant organogenesis. However, understanding the complex feedback signaling network that involves at least 29 proteins in Arabidopsis in the dynamic context remains a significant challenge. To address this, we transplanted an auxin-responsive feedback circuit responsible for plant organogenesis into yeast. By generating dynamic microfluidic conditions controlling gene expression, protein degradation, and binding affinity of auxin response factors to DNA, we illuminate feedback signal processing principles in hormone-driven gene expression. In particular, we recorded the regulatory mode shift between stimuli counting and rapid signal integration that is context-dependent. Overall, our study offers mechanistic insights into dynamic auxin response interplay trackable by synthetic gene circuits, thereby offering instructions for engineering plant architecture.

Auxin, pivotal for plant organogenesis (1), drives interactions between Auxin/ INDOLE-3-ACETIC ACID (Aux/IAA) (2) repressors and TRANSPORT INHIBITOR RESPONSE 1/AUXIN SIGNALLING F-BOX PROTEIN (TIR1/AFB) (3, 4) auxin receptors in the nucleus, promoting AUX/IAA degradation and AUXIN RESPONSE FACTOR (ARF)-mediated activation of many genes including AUX/IAAs, thereby fueling a negative feedback (5, 6). ARFs contain domains for DNA binding, transactivation, and oligomerization, which also appear on AUX/IAA proteins for homo- and hetero-oligomerization (7–9). This intricate feedback loop, involving 29 ARF and AUX/IAA members, governs *Arabidopsis thaliana* organogenesis (10, 11). Despite extensive molecular understanding, feedback effects on AUX/IAA degradation, gene expression, and ARF-DNA kinetics in dynamic contexts remain unclear.

Recreating auxin signaling in yeast (*Saccharomyces cerevisiae*), lacking plant components, unveiled constitutive AUX/IAA-ARF interactions (12). However, the core feedback layer's absence and its dynamic performance remain unexplored. The central enigma revolves around how auxin response components mutually influence and shape responses in dynamic conditions.

## Results

**Fig. 1.**
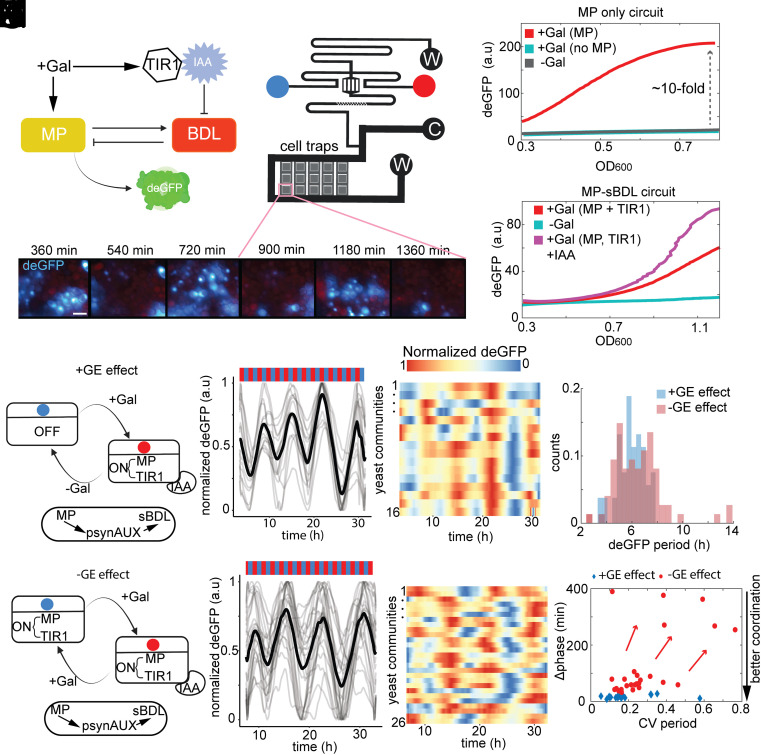
Pronounced effect of gene expression on the coordination of auxin responses in a dynamic context. (*A*) Schematic of yeast-based reconstituted MP-BDL circuit. Auxin response is monitored through destabilized eGFP (deGFP), induced by galactose-driven MP and TIR1 expression. (*B*) Microfluidic device with switchable media reservoirs generating two dynamic conditions (panels *F* and *J*). (*C*) deGFP fluorescence recorded over hours in cell traps (500 × 500 µm), zoom on the cell trap populated by yeast cells. (*D*) PsynAUX promoter leakiness test in constitutive presence (+Gal) or absence of MP (−Gal). (*E*) Time-course deGFP fluorescence in batch cultures (fixed condition) with or without IAA. Trends with 95% CIs collected from three independent replicates. (*F*–*H*) Microfluidic experiments, Scenario 1 (+GE): galactose-driven MP/TIR1, auxin-triggered sBDL degradation (*G*), shows coordinated auxin response in dozens of yeast populations (n = 16) (*H*). Red and blue dots and bars denote +IAA and −IAA condition, respectively. (*I*) Cumulative deGFP periods in +GE (blue) and −GE (red) scenarios. (*J*−*L*) Scenario 2 (-GE): constitutive MP/TIR1 expression, auxin-pulsed sBDL degradation (*K*), yields less coordinated auxin response in yeast populations (n = 26) (*L*). (*M*) Degree of auxin response coordination (Δphase) versus coefficient of variation (CV) reflecting period noise level indicates better coordination of auxin response in GE scenario compared to NGE scenario (arrows show coordination loss).

We utilized a synthetic biology approach to investigate the feedback mechanism in auxin signaling regulation, focusing on engineering synthetic auxin-responsive circuits. These circuits featured two key regulators of organogenesis in *A. thaliana*, ARF5 Monopteros (MP) and IAA12 Bodenlos (BDL) (13), integrated into a system mirroring the plant's circuit architecture controlling organ development (Fig. 1*A*). We manipulated MP gene expression using galactose and BDL degradation rates with auxin to probe the auxin feedback mechanism's response to controlled changes in MP and BDL levels.

Our synthetic circuit design began with the creation of an auxin-responsive promoter, psynAUX, which was created by fusing inverted repeats of MP-bound cis-regulatory elements (14) with a minimal yeast promoter. To enhance the auxin-response reporter's dynamic range, we employed the psynAUX promoter to drive a destabilized green fluorescent reporter (dEGFP) (15). Time-course fluorescence assays in batch yeast cultures with constant galactose presence demonstrated a nearly 10-fold MP-dependent increase in dEGFP fluorescence, with negligible leakage under control conditions (Fig. 1*D*).

Given previous indications of inadequate repression by Aux-IAA repressors alone in yeast (12, 16), likely due to suboptimal Histone deacetylase (HDAC) recruitment (17), we engineered an active repressor synthetic BODENLOS (sBDL) by fusing the yeast repression domain from the SSN6 repressor (18) to BDL. SSN6 interacts with TUP1 (19), akin to plant TPL that interacts with BDL (20). This linkage allowed coupling the synthetic MP-sBDL circuit with yeast HDAC machinery, which has evolutionary conservation with plants (20). Introducing a negative feedback loop, we expressed sBDL repressor from the psynAUX promoter, resulting in clear repression (Fig. 1*E*) compared to the control lacking sBDL (Fig. 1*D*) in batch culture experiments. To enable auxin-controlled sBDL degradation, we expressed *A. thaliana* TIR1 auxin receptor under a galactose-inducible promoter. Time-course assays in yeast cultures demonstrated that this synthetic circuit responded to auxin by releasing MP from repression (Fig. 1*E*), confirming responsiveness to auxin and MP dependence.

Next, we created a dynamic context, using time-lapse live cell imaging in microfluidic devices (Fig. 1 *B* and *C*) (21, 22). We explored two dynamic conditions: Scenario 1 involved coordinated gene expression (+GE) effects of MP and TIR1 with auxin-controlled sBDL degradation (Fig. 1*F*), and Scenario 2 simulated constant MP and TIR1 expression with periodic auxin stimulation that refers to lack of gene expression effect (−GE) (Fig. 1*J*). The +GE effect produced a robust response across yeast populations (n = 16 populations, each with >1k cells), with 6-h periods for each 2-h cycle of auxin pulses (Fig. 1 *G* and *H* and Movie S1). Constitutive MP and TIR1 expression yielded a broader period distribution (n = 26), with some periods extending up to 10 to 14 h (Fig. 1 *I* and *M*). Differences in deGFP peak timings (Δphase) recorded in all yeast populations were smaller for the +GE case, indicating that +GE together with auxin-regulated degradation can coordinate auxin responses through dynamic changes in circuit component availability (Fig. 1*M*). Both conditions generated dEGFP reporter response once per three auxin pulsing cycles (Fig. 1*I*); intriguing slower bursts are likely linked to repressor degradation delays and negative feedback loop effects.

In summary, +GE effect with auxin-mediated repressor degradation coordinated auxin response at the population level in a dynamic context. This insight may have implications for tailoring auxin responses via modulation of auxin signaling component availability for precise postembryonic organ patterning in planta (1, 23).

**Fig. 2.**
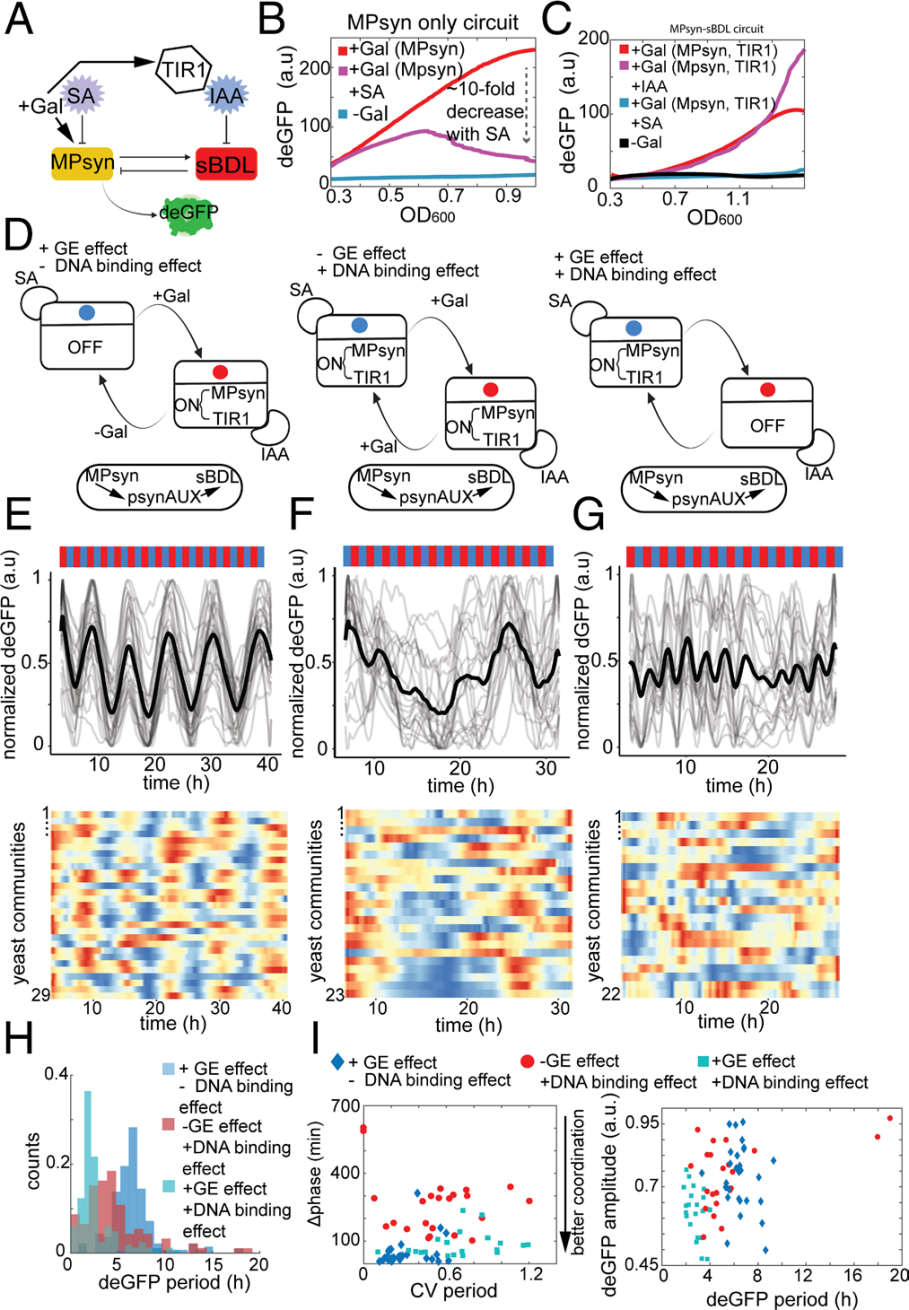
Regulated MP-DNA association and gene expression jointly control the speed and coordination of auxin responses to dynamic auxin pulses at whole population level. (*A*) Schematic of synthetic auxin response circuit with MP DNA binding control (MPsyn) by salicylic acid (SA). This setup enables independent control of SA-dependent MP-DNA association, MP/TIR1 gene expression by galactose, and sBDL degradation by auxin. (*B* and *C*) Time course of dEGFP fluorescence vs. cell density (OD_600_) in MPsyn alone and MPsyn-sBDL circuits with or without IAA and/or SA in batch cultures with fixed conditions. Trends with 95% CIs collected from three independent replicates. (*D*–*G*) Microfluidic experiments testing effects of DNA binding, GE, and sBDL degradation effects on auxin responsiveness are presented with schematics (*D*). +GE effect matches Fig. 1*G* (n = 29) (*E*); −GE and +DNA binding effects (n = 23) (*F*) and +GE and +DNA binding effects (n = 22) show faster dEGFP reporter pulses (*G*). (*H*–*I*), Cumulative deGFP periods (*H*) shift progressively, indicating regulated DNAbinding’s influence. (*I*) Regulated DNA binding controls auxin response speedand approximate noise level (CV period) (left panel), and mean amplitudes vs. mean periods show antithetic relation (right panel).

Our auxin-responsive circuit precisely counted auxin stimuli, but neither auxin-based degradation nor gene expression alone controlled the system's response timing. We speculated that regulated MP-DNA association could influence this feature inspired by our recent work (21). To test, we introduced regulated DNA binding by creating a chimeric MPsyn activator (Fig. 2*A*), swapping MP DNA binding domain for bacterial transcription factor from Mar family that is inhibited by salicylate (21, 24, 25). The new circuit was activated by MPsyn alone and was sensitive to both salicylate and auxin in fixed-condition batch cultures (Fig. 2 *B* and *C*). In microfluidic environment, +GE effect alone yielded similar responses to original circuits, with 6-h periods (Fig. 2 *E* and *H* and Movie S3). However, -GE effect combined with +DNA binding regulation led to much noisier yet faster responses compared to the +GE alone (Fig. 2 *F* and *H* and Movie S4). The combination of +GE and +DNA binding significantly expedited auxin response timing maintaining the response coordination (Fig. 2 *H* and *I* and Movie S5). An auxin response timing correlated negatively with auxin response amplitude due to DNA binding regulation (Fig. 2*I*, right panel).

These findings suggest that +DNA binding kinetics control auxin response pace but are not sufficient to provide coordination across cell populations. Concurrently, +GE effect maintains response coordination in a dynamic context, whereas auxin-mediated degradation enables system resetting. All effects contribute to auxin overall response characteristics and could govern distinct spatiotemporal outcomes. Thus, insights from our synthetic systems may aid in understanding complex auxin response dynamics in planta.

## Discussion

In conclusion, regulated DNA binding and availability of signaling components play significant roles in tailoring auxin response dynamics in our synthetic system, whereas auxin-regulated repressor degradation helps to maintain coordinated auxin responses at the population level. This offers insights into how local dynamic cues alter auxin signaling component availability and kinetics, influencing plant developmental contexts. Our quantitative synthetic biology in yeast remains a powerful tool for studying plant hormone responses quantitatively and spatiotemporally in controlled microfluidic conditions. This strategy can explore complex auxin signaling circuits and other plant hormonal pathways, sidestepping regulatory network complexities. Our study provides insights into hormone responses in plant gene circuits, offering a valuable strategy for plant hormone research.

## Materials and Methods

Full methods are presented in *SI Appendix*. An isothermal single-tube Gibson assembly (26) was used to assemble amplified DNA fragments and build synthetic auxin-responsive circuits. Sequences of circuit components are provided in Dataset S1. Microfluidics devices and controllable inflow system were designed based on our previous works (21, 22).

## Supplementary Material

Appendix 01 (PDF)

Dataset S01 (XLSX)

Movie S1. **+GE effect impact on dynamics of auxin response circuit**. Example time-lapse imaging of living yeast populations in the microfluidic device. Color coding as in Fig. 1. Left panel shows Differential interference contrast (DIC) image and the right panel show cells in red (constitutive mCherry expression) and deGFP is shown in cyan. Related to Fig. 1G, H.

Movie S2. **-GE effect and auxin responsiveness in the microfluidic environment**. Example time-lapse imaging of living yeast populations in the microfluidic device. Color coding as in Fig. 1. Left panel shows Differential interference contrast (DIC) image and the right panel show cells in red (constitutive mCherry expression) and deGFP is shown in cyan. Related to Fig. 1K, L.

Movie S3. **+GE effect alone in MarR-MP system**. Example time-lapse imaging of living yeast populations in the microfluidic device. Color coding as in Fig. 1. Left panel shows Differential interference contrast (DIC) image and the right panel show cells in red (constitutive mCherry expression) and deGFP is shown in cyan. Related to Fig. 2E.

Movie S4. **-GE and -DNA binding scenario and auxin responsiveness in MarR-MP circuit**. Example time-lapse imaging of living yeast populations in the microfluidic device. Color coding as in Fig. 1. Left panel shows Differential interference contrast (DIC) image and the right panel show cells in red (constitutive mCherry expression) and deGFP is shown in cyan. Related to Fig. 2F.

Movie S5. **Combination of GE and regulated DNA binding effects on auxin responsiveness in MarR-MP system**. Example time-lapse imaging of living yeast populations in the microfluidic device. Color coding as in Fig. 1. Left panel shows Differential interference contrast (DIC) image and the right panel show cells in red (constitutive mCherry expression) and deGFP is shown in cyan. Related to Fig. 2G.

## Data Availability

All study data are included in the article and/or supporting information.
