# Supplementary material for: Dynamic context-dependent regulation of auxin feedback signaling in synthetic gene circuits
Source: Proc Natl Acad Sci U S A. 2023 Oct 9;120(42):e2309007120. doi: 10.1073/pnas.2309007120 (PMC10589675; doi:10.1073/pnas.2309007120)
Supplement: Supplementary file 1 — Appendix 01 (PDF) [file pnas.2309007120.sapp.pdf]

Supporting Information for

Dynamic context-dependent regulation of auxin feedback signaling in synthetic gene circuits

Mersia Avdovic[1], Mario Garcia-Navarette [1], Diego Ruiz-Sanchis[1], Krzysztof Wabnik[1,*]

[1] Centro de Biotecnologia y Genomica de Plantas (Universidad Politécnica de Madrid – Instituto Nacional de Investigación y Tecnologıa Agraria y Alimentaria), Autopista M-40, Km 38 – 28223 Pozuelo de Alarcón, Spain

Email: k.wabnik@upm.es

This PDF file includes:

Supporting Methods
Legends for Movies S1 to S5

Other supporting materials for this manuscript include the following:

Movies S1 to S5

Dataset 1

Supporting Methods

Vector constructions and DNA cloning. The vectors were designed and prepared as follows: The psynAUX promoter was created with two inverted repeats of auxin response motifs, TGTCTC and TGTCGG, located upstream of a minimal TATA-box. The promoter was synthesized by Integrated DNA Technologies and then cloned into the pGADT7 vector (Takara Bio Inc.). Histidine auxotrophic selection marker upstream of the deGFP reporter gene. The full-length MP gene was expressed from the galactose-inducible pGAL7 promoter and then cloned into the pGADT7 vector from Takara Bio Inc. The vector contained a histidine auxotrophic selection marker. sBDL was created by fusing the full-length BDL/IAA12 gene to the C-terminal part of the SSN6 repression domain (aa 1-166). This construct was then cloned into the pGADT7 vector from Takara Bio Inc. The vector contained a uracil selection marker. MPsyn was designed by fusing the full-length MarR transcription repressor to MP, but with the DNA binding domain (aa 348-902) removed using a flexible linker. The MPsyn construct was driven by the galactose-inducible pGAL7 promoter and then cloned into the pGADT7 vector from Takara Bio Inc. The vector contained a leucine selection marker. For specific DNA sequences, please refer to Dataset 1.

Assembly of DNA fragments. An isothermal single-tube Gibson assembly (1) method was used to seamless cloning of the DNA fragments. Synthetic fragments with annealing and overlapping regions were designed using Benchling (https://www.benchling.com/) and IDT Codon Optimization tool (Integrated DNA Technologies). The annealing regions were made complementary to the terminal sequences of the target DNA fragment. The appropriate DNA fragments, amplified through PCR, were mixed in a 1:1 ratio. This mixture was then combined with 2.5 µL of Gibson mix and incubated at 50 °C for an hour.

Transformation and validation of assembled DNA. The assembled DNA product was transformed into E. coli DH5α cells using a heat-shock method: unfrozen cells were thawed in ice with the Gibson product for 30 minutes. Then, the cells were subjected to a heat shock at 42 °C for 45 seconds, followed by recovery in LB Broth Miller (Formedium, UK) at 37 °C for 45 minutes. The transformed cells were then plated on LB 2% agar plates containing 100 µg/mL carbenicillin (Cb) and incubated overnight at 37°C. The next day, colonies were selected from the plate, grown in liquid LB with 100 µg/mL Carbenicilin overnight, and plasmids were purified using the GeneJET Plasmid Miniprep Kit. The purified plasmids were then validated through a sequencing service (Macrogen Spain).

Transformation of yeast strains. The plasmids obtained using Gibson assembly cloning were transformed in competent S. cerevisiae BY4741 (his3Δ1 leu2Δ0 met15Δ0 ura3Δ0) expressing constitutive mCherry marker (2) and full-length TIR1 auxin receptor (3) under the pGAL1 promoter. The transformation was performed using the Frozen-EZ Yeast Transformation II Kit (Zymo Research, USA) according to the manufacturer's instructions. The yeast strains were transformed with up to three plasmids, each containing a selection marker for the complementation of uracil, histidine, or leucine auxotrophy.

Yeast strain selection. The yeast constructions were incubated at 30°C for three days on plates containing 2% glucose. Throughout the process, minimal drop-out media was used to prevent plasmid loss.

Multiwell plate activity measurements. Circuitswere analyzed under static conditions using a microplate reader fluorescence assays. Time-lapse measurements of OD600 (absorbance at 600nm wavelength) and fluorescence intensity (λEx = 488 nm; λEm = 515 nm) were performed

every 10 minutes over 24 hours at 30 °C. The activation of the system was analyzed by recording a time-lapse profile of deGFP fluorescence. To accomplish this, overnight cultures were diluted to a total OD600 of 0.1. Additional compounds, in this case, salicylic acid (SA 250 µM), galactose (Gal7 0.25%), and indole-3-acetic acid (10 µM IAA, auxin) were added at this point. Aliquots of 200 µL were pipetted from these diluted cultures with a different gradient concentration of these compounds to a 96-well plate. Since the constructs of our system were placed under galactose-inducible promoters, the glucose and sucrose conditions were included as negative controls. Fluorescence and OD_{600} measurements obtained from the plate reader were exported to tabular format and processed using R (R Core Team, 2017) and the package dplyr. Time-course plots were produced using the package ggplot2. This allows for the visualization of the dynamic changes in fluorescence and absorbance over time, as well as the comparison between different conditions such as different concentrations of the added compounds.

Assembly of microfluidics devices. All engineered strains were analyzed using inverted epifluorescence microscopy in 72-hour time-lapse experiments in a microfluidic chip under dynamically generated conditions as described previously (2). To create molds for microfluidic chips, we used InkScape to design the molds and printed them onto plastic sheets using a monochrome laser printer at 1200 dpi resolution. By controlling the density of ink deposition, we were able to regulate the height of the features. The plastic wafers were then cut to size and placed in a thermal oven set to 160°C to shrink by one-third of their original size. After baking for 10 minutes to smooth and harden the ink, the molds were cleaned with soap and rinsed with isopropanol and DDI water before being dried with a nitrogen gun and secured with Scotch tape. The cleaned molds were then placed into a clean 90mm Petri dish and coated with Sylgard 184 Polydimethylsiloxane (PDMS) mixed in a ratio of 10:1 with elastomer. The PDMS was degassed in a vacuum cleaner for approximately 15 minutes and cured at 60 °C overnight. The next day, the molds were removed from the PDMS, and the chips were cut to their final shape. The ports were opened using a 0.7mm biopsy puncher (World Precision Instruments). At the same time, cover glasses were cleaned in a sonic bath, rinsed in ethanol and water, and finally cleaned with a nitrogen gun. Before bonding the cover glasses with the chip, they were exposed to oxygen plasma using a Corona SB plasma treater (ElectroTechnics Model BD-20AC Hand-Hld Laboratory Corona Treatar) for about 20 seconds. Finally, the cover glasses and chips were bound together and placed in an oven at 60°C overnight.

Testing and optimizing the microfluidic mixer module for reliable mixing. To calibrate the mixer, we stained one of the two inputs with Rhodamine B 0.001% (w/v) and prepared a pair of 60mL syringes with either 20mL of water or 20mL of water + dye. Three 50mL falcon tubes filled with 20mL of water were connected to the waste and cell loading ports, and the syringes and falcon tubes were set on linear actuators that control pressure in the microfluidic device via customized software. Before connecting Tygon microbore tubing 0.020" x 0.060" OD (ColePalmer Inc.), the microfluidic device was vacuumed for at least 20 minutes to remove air bubbles and facilitate device wetting. One syringe was connected, and then the other syringes were plugged sequentially once the liquid reached and filled the ports. Switching between inputs was regulated by the gravity-aided hydrostatic pressure through changing the relative height of the syringes. Increasing the height of input media 1 (M1, SA 250 µM) over input media 2 (M2, IAA 10 µM) produced the pressure difference in the mixing module, filling the microfluidic chip with 100% M1 and 0% M2 at maximum height differences. The syringe positions were adjusted with 0.1 mm precision by changing height to obtain highly efficient mixing between 0% and 100%. Excessive flow was diverted towards an auxiliary waste output (W1) that releases the pressure from the mixer. Flow was then introduced into the second chaotic mixer to ensure rapid switching of conditions. We repeated the calibration process at least three times with three independent microfluidic devices. The maximum and minimum height settings varied by only a few millimeters, with an average of 610 mm and 390 mm, respectively. To avoid cross-contamination of media inputs, we used averaged measures. All three replicates showed exact 50% mixing for the 500 mm settings.

Loading of cells and media environmental control. Freshly cultivated strains were grown overnight in minimal low fluorescence yeast media with 2% sucrose as a carbon source. The next day, the yeast culture was diluted 10 times into a falcon container and the OD was approximately 0.4, making it easy to introduce the yeast culture into the chip. Under sterile conditions, tubing lines were cleaned with ethanol and connected to syringes containing fresh media and to the falcon container, which was used as the output container for introducing fresh yeast culture. Additionally, after cleaning the lines, media were prepared in 60mL syringes using 25mL of volume to achieve the desired concentration of inducing media by adding certain hormones into the system's inputs. The same process of cleaning was done for the outputs of the system, and 10mL of DDI water was introduced. Before plugging, the microfluidics chip was vacuumed for at least 20 minutes to facilitate the loading process. The input syringes were placed in a height control device to regulate the flow by adjusting their relative height compared to the output containers. The tubing lines were then plugged into the chip one by one, ensuring that the media's flow reached all features of the chip and no air bubbles were trapped. The tube with the cells was lifted until the cells began to flow and got captured via microvalves into the chip at a significant rate and started seeding the trapping region. Once 10-20 cells were retained in each trap, the flow from the loading cell port was reverted by decreasing the height of the tube to prevent the entry of bubbles during the experiment.

Time-lapse microscopy in a microfluidic device and image analysis. The imaging was carried out using an automated inverted Leica DMi8 fluorescence microscope with a Hamamatsu Orca Flash V3 camera. Each trap was captured every 10 minutes in three different channels: differential interference contrast (DIC), GFP (λEx = 488 nm; λEm = 515 nm), and mCherry (λEx = 583 nm; λEm = 610 nm). The acquisition of images was controlled by the software μManager (https://micro-manager.org/). A 40x dry objective (Leica, Germany) was used to capture the images with a CoolLed pE600 LED source and a standard epifluorescent filter set (Chroma, USA). The multichannel image sequences were subsequently processed using the Fiji software (https://imagej.net/Fiji). A Constitutively expressed mCherry marker was used to identify exponentially growing cells and used to derive normalised dEGFP fluorescence: dead or non-growing individuals were discarded by correcting dEGFP signal according to the formula $dEGFP/(dEGFP + mCherry)$. Each image was divided into 25 regions of interest (ROIs) and analysed separately to isolate regions where cells were actively growing and could be tracked over time. The posterior analysis was done with custom R-studio scripts. Firstly, raw data were detrended using the detrend function from “pracma” R-studio v4.0.3 package and then smoothed with Savitzky–Golay Smoothing function (savgol), from the same package, with a filter length of 15 was applied and the signal was normalised between 0 and 1 to generate heatmaps across cell traps. Amplitudes were calculated with find peaks within the Process Data using the “findpeaks” function from “pracma” R package with nups and ndowns of 6, and periods were calculated by calculating distances between successive dEGFP peaks. Phase difference (∆phase) was calculated by comparing time differences of successive deGFP peaks between yeast communities (cell traps) in microfluidic devices to derive a relative measure of inter-community gene expression coordination ($n > 15$, ~15,000 cells). Coefficient of variation (CV) represents ration of standard deviation to mean of cumulative deGFP fluorescence periods in all reported cell traps.

Legends for Movies S1 to S5

Movie S1. +GE effect impact on dynamics of auxin response circuit. Example time-lapse imaging of living yeast populations in the microfluidic device. Color coding as in Fig. 1. Left panel shows Differential interference contrast (DIC) image and the right panel show cells in red (constitutive mCherry expression) and deGFP is shown in cyan. Related to Fig. 1G, H.

Movie S2. -GE effect and auxin responsiveness in the microfluidic environment. Example time-lapse imaging of living yeast populations in the microfluidic device. Color coding as in Fig. 1. Left panel shows Differential interference contrast (DIC) image and the right panel show cells in red (constitutive mCherry expression) and deGFP is shown in cyan. Related to Fig. 1K, L.

Movie S3. +GE effect alone in MarR-MP system. Example time-lapse imaging of living yeast populations in the microfluidic device. Color coding as in Fig. 1. Left panel shows Differential interference contrast (DIC) image and the right panel show cells in red (constitutive mCherry expression) and deGFP is shown in cyan. Related to Fig. 2E.

Movie S4. -GE and -DNA binding scenario and auxin responsiveness in MarR-MP circuit. Example time-lapse imaging of living yeast populations in the microfluidic device. Color coding as in Fig. 1. Left panel shows Differential interference contrast (DIC) image and the right panel show cells in red (constitutive mCherry expression) and deGFP is shown in cyan. Related to Fig. 2F.

Movie S5. Combination of GE and regulated DNA binding effects on auxin responsiveness in MarR-MP system. Example time-lapse imaging of living yeast populations in the microfluidic device. Color coding as in Fig. 1. Left panel shows Differential interference contrast (DIC) image and the right panel show cells in red (constitutive mCherry expression) and deGFP is shown in cyan. Related to Fig. 2G.

Supplemental bibliography

1. Gibson DG, Young L, Chuang RY, Venter JC, Hutchison CA, Smith HO. (2009). Enzymatic assembly of DNA molecules up to several hundred kilobases. Nature Methods, 6(5), 343–345.

2. Pérez-García S. et al. (2021) Synchronization of gene expression across eukaryotic communities through chemical rhythms. Nat. Commun. 12, 4017

3. Yu H, Moss BL, Jang SS, Prigge M, Klavins E, Nemhauser JL, Estelle M. Mutations in the TIR1 auxin receptor that increase affinity for auxin/indole-3-acetic acid proteins result in auxin hypersensitivity.
